# Novel presentation of acalvaria with clavicular absence: A case report

**DOI:** 10.1097/MD.0000000000040399

**Published:** 2024-11-08

**Authors:** Wasef Alhroub, Maaweya Jabareen, Mousa Humeedat, Mosaikah Anati, Bashar Qanaeer, Asrar Alhroub

**Affiliations:** a College of Medicine, Hebron University, Hebron, Palestine; b Pediatric Surgery, Hebron Governmental Hospital, Hebron, Palestine.

**Keywords:** acalvaria, cranial vault, neural tube defects, skull vault

## Abstract

**Rationale:**

Acalvaria is an exceptionally rare congenital disorder marked by the absence of flat bones of the cranial vault, dura mater, and associated muscles, while the facial bones and base of the skull remain intact. Typically, the central nervous system is unaffected. Due to their extreme rarity, reported cases in the literature are infrequent. This condition often results in fatalities, as newborns with this anomaly generally have short life expectancies. However, there are a few documented cases of prolonged survival.

**Patient concerns:**

A 1-day-old full-term Palestinian male born to first-cousin parents at 37 + 6 weeks gestational age presented with an excessively soft skull without scalp abnormalities. The mother experienced polyhydramnios during the last 7 weeks of pregnancy.

**Diagnoses:**

Head ultrasound and X-rays confirmed the absence of skull vault bones and clavicles with normal facial bones. A CT scan showed well-formed brain structures and foci of hemorrhage in the left frontal lobe.

**Interventions:**

Supportive care was the primary management approach. The patient received comprehensive care in the neonatal intensive care unit, with a focus on stabilization and monitoring.

**Outcome:**

After 3 weeks in the neonatal intensive care unit, the patient showed normal feeding and function, but the prognosis remained poor. The patient’s family was informed about the poor prognosis.

**Lessons:**

This case reveals a unique combination of acalvaria and absent clavicles. Early antenatal diagnosis is essential but was delayed here. More research is needed to understand and improve the diagnosis of these conditions.

## 1. Introduction

The calvaria, or skull vault, is the membranous portion of the neurocranium. It primarily consists of flat bones, including the paired frontal and parietal bones, the squamous parts of the temporal bones, and the interparietal part of the occipital bone.^[1]^ Aplasia of the membranous neurocranium and dura mater, with intact chondrocranium (skull base, facial bones, scalp, and cerebral hemispheres), is known as acalvaria.^[2]^

The pathophysiology of acalvaria is yet unknown; however, a post-neurulation defect is believed to be the cause.^[3]^ A post-neurulation defect is 1 of the 2 types of neural tube defects (NTDs) that affect the brain and spinal cord at different points during pregnancy. NTDs can be divided into open (neurulation defects) and closed (post-neurulation defects). Open defects are much more frequent than closed defects, and they show the exposed nerve tissue as anencephaly and meningomyelocele. Post-neurulation NTDs are less common and have skin coverings such as encephalocele, which can be misdiagnosed with acalvaria on ultrasonography.^[4]^

Acalvaria is typically considered a fatal anomaly. This review aims to highlight the unusual clinical presentations that may be present in this rare condition and focuses on the importance of early antenatal diagnosis in enabling parents to make informed decisions about the continuation of the pregnancy.

## 2. Case presentation

A 1-day-old full-term Palestinian male was presented with an excessively soft skull without scalp abnormalities. The patient’s parents are first cousins, and he was delivered vaginally at 37 + 6 weeks gestational age to G6P6A0 mother.

Antenatally, the mother received regular follow-up care, with polyhydramnios observed in the last 7 weeks of pregnancy. However, a detailed ultrasound was not performed. There was no history of fever, urinary tract infection, or rash during pregnancy. The mother received intermittent folic acid supplementation after the first 9 weeks of pregnancy, along with iron and calcium supplementation, with no other medications. There was no history of smoking, alcohol consumption, or radiation exposure. Additionally, there was no family history of genetic disease or congenital malformations.

Upon examination, the neonate’s weight was 2700 g, his length was 51 cm, and his head circumference was 34 cm. Apgar scores were 7 at 1 minute and 8 at 5 minutes. Physical examination revealed normal skull and facial structures, but palpation revealed the absence of frontal, temporal, and bilateral parietal bones, with normal occipital and facial bones. The scalp and skin appeared normal and covered the bony defect. Clavicles were not palpable in the chest. Lung examination revealed typical bilateral and equal breath sounds. A cardiovascular examination showed clear first and second heart sounds without murmurs. On neurological evaluation, the Moro reflex was present and symmetrical, with normal peripheral tone and good sucking.

**Figure 1.**
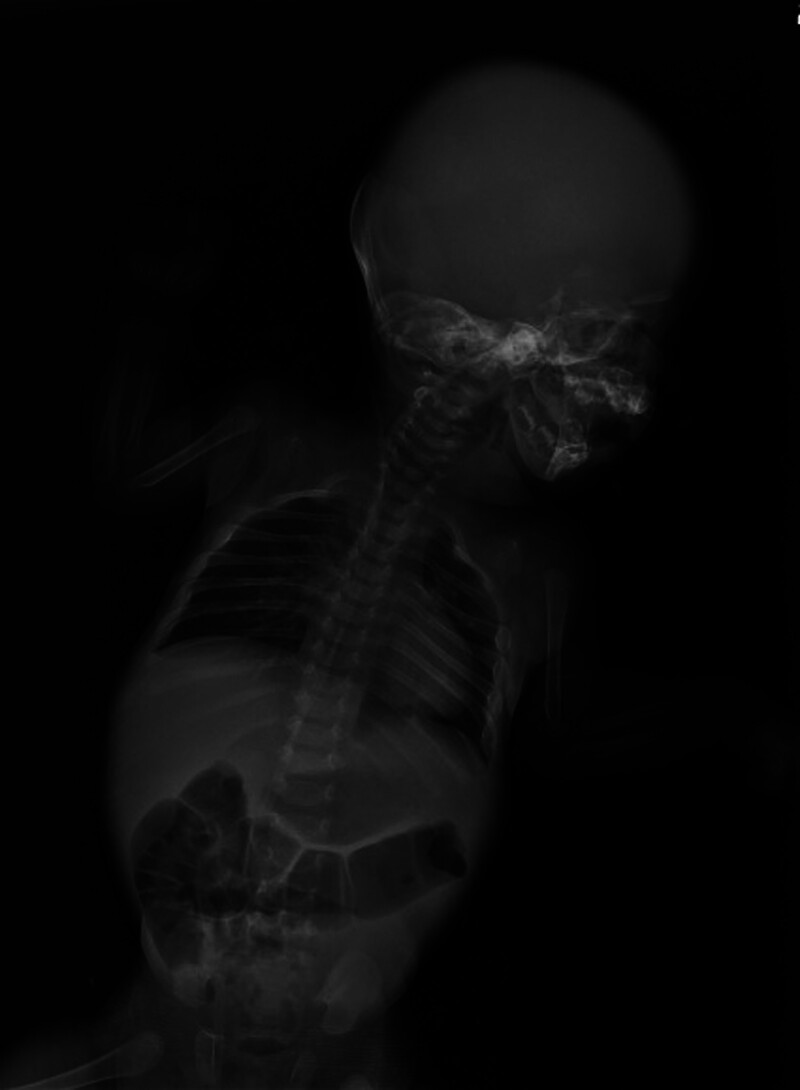
Head X-ray showed the absence of frontal, temporal, and bilateral parietal bones.

**Figure 2.**
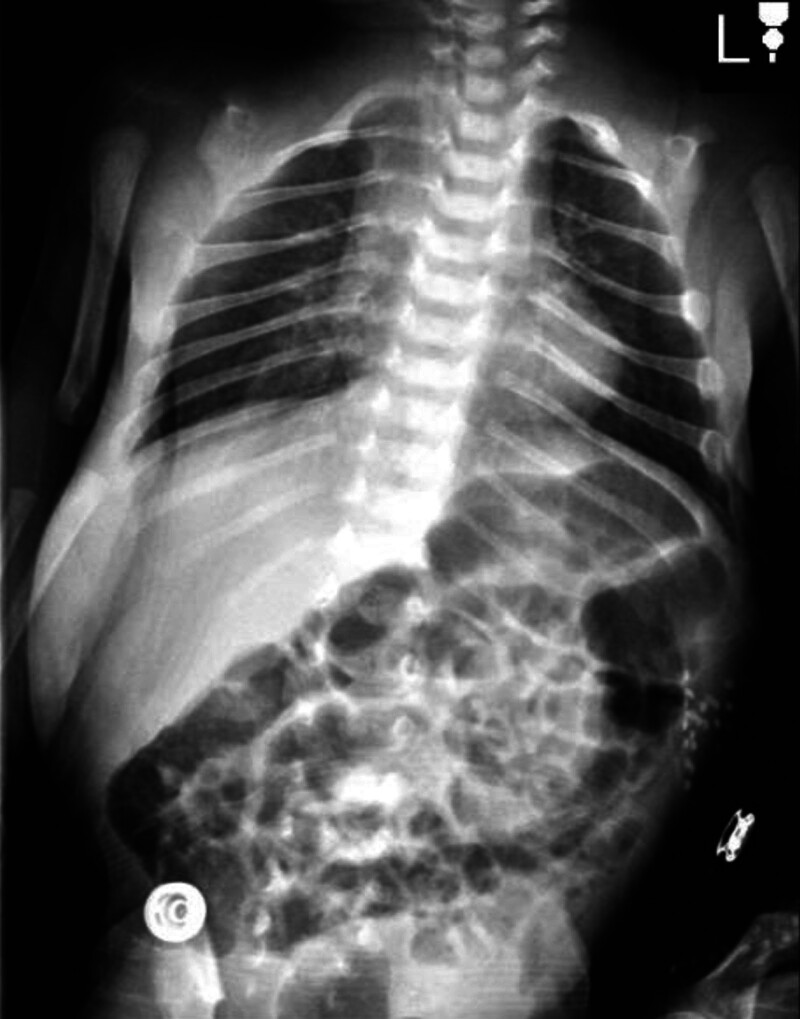
Chest and abdomen X-ray showed the absence of both clavicles.

**Figure 3.**
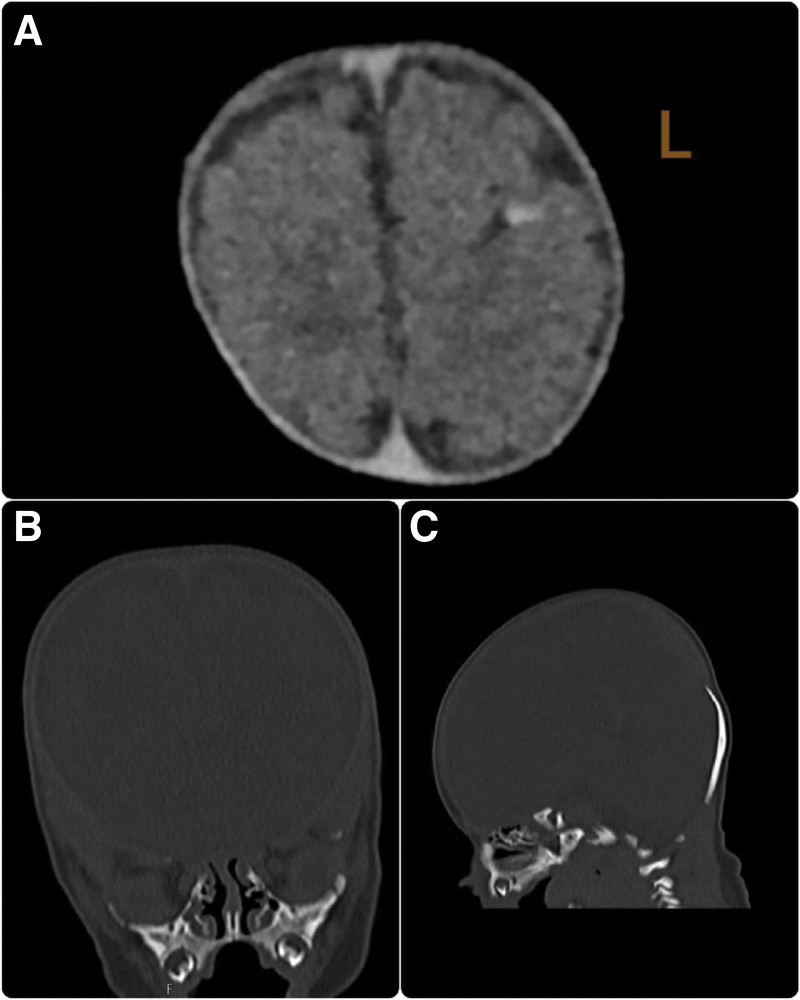
(A) Axial CT scan without IV contrast of the brain window showed foci of parenchymal hemorrhage at the left frontal lobe. (B) Coronal CT scan without IV contrast showed normal facial bones with absent frontal and parietal bones. (C) Coronal CT scan without IV contrast showed absent all calvarium bones with preservation of the occipital bone.

A head ultrasound was performed and revealed the absence of skull vault bones and visible facial bones. The brain examination shows well-formed brain lobes with normal sulci and gyri, as well as a normal corpus callosum and posterior fossa. There is no evidence of hydrocephalus or brain hemorrhage. X-rays of the skull and chest confirmed the absence of frontal, temporal, and bilateral parietal bones (Fig. 1), as well as the absence of both clavicles (Fig. 2). A computed tomography scan of the brain showed the absence of all calvarial bones with preservation of the occipital and facial bones. Additionally, axial sections in the brain window showed foci of parenchymal hemorrhage in the left frontal lobe (Fig. 3A–C).

The patient was admitted to the neonatal intensive care unit for a comprehensive assessment. Initially, formula feeding was provided, which was later transitioned to breastfeeding. The patient demonstrated normal gastrointestinal function, passed meconium, and maintained regular urinary output. The neonate remained in the neonatal intensive care unit due to physiological jaundice and thrombocytopenia.

At 3 weeks of age, the infant was active, weighing 2800 g with a head circumference of 35 cm. Examination revealed normal-colored sclerae and protruded eyes due to a lack of frontal bone, along with hypertelorism. Mongolian spots were noted on the back; the limbs and chest appeared normal. Despite these observations, the prognosis remained poor, and the family was informed of this.

## 3. Discussion

Acalvaria is an exceedingly rare congenital malformation characterized by the absence of cranial vault bones, the dura mater, and the associated muscles.^[3,5,6]^ While the central nervous system and cranial contents typically remain unaffected, neuropathological defects, to some extent, have been reported in a few cases.^[3,5]^ It has been associated with a range of congenital anomalies, including holoprosencephaly, hydrocephalus, micropolygyria, cardiac anomalies, omphalocele, hypertelorism, cleft lip and palate, renal tubular dysgenesis, hexadactyly, clubfoot, and congenital medulloblastoma.^[3,5–7]^ The presence of these associations underscores the importance of comprehensive clinical evaluation. Such assessments are crucial for accurately determining the prognosis and predicting the final outcome in these rare cases.

The cause of acalvaria remains unknown, and researchers are actively studying its origins. Current literature suggests that the condition may stem from defects after the closure of the anterior neural pore around the 4th week of embryonic development. During normal development, mesenchymal tissue should migrate beneath the ectoderm to form muscle and bone, while the ectoderm becomes skin and scalp. When this process is disrupted, the result can be the absence of flat bones in the skull and associated muscles, while the brain remains covered by skin. Despite these abnormalities, the base of the skull usually develops normally, as it originates from the cartilaginous part of the neurocranium.^[3,5]^

Other theories suggest that acalvaria could result from primary non-closure of the neural tube, while some researchers believe it may be part of a spectrum of conditions that includes anencephaly.^[3,5,8]^ Another theory proposes that acalvaria is an early embryonic event triggered by a localized hypoxic insult. This disturbance is believed to interfere with neural crest differentiation, eventually resulting in the absence of the neurocranium.^[9,10]^

In our case, the absence of frontal, temporal, and parietal bones, combined with an intact skull base, facial structure, and hypertelorism, reflects abnormalities commonly seen in acalvaria.^[3,5–7,11,12]^ However, the intact occipital bone observed in our case is an unusual association, and only 1 reported case has documented this presentation.^[13]^

Additionally, our case presents a unique association of acalvaria with the absence of clavicles, a combination that, to our knowledge, has not been previously reported in the literature. This novel presentation raises intriguing questions about the underlying developmental mechanisms. Acalvaria is thought to result from a failure of the mesenchymal tissue beneath the ectoderm to form bone. The concurrent absence of clavicles suggests a broader disruption of skeletal development, possibly implicating signaling pathways involved in both cranial and appendicular skeletal formation.

There have been no reported associations between acalvaria and chromosomal abnormalities, and the majority of cases are identified as sporadic occurrences. Ultrasonography can serve as a valuable tool for the antenatal diagnosis of cranial bone abnormalities, particularly during the second trimester of pregnancy, once the mineralization of the skull bones is complete.^[14]^ The key sonographic difference distinguishing acalvaria from anencephaly is the presence of normal cerebral hemispheres in acalvaria cases. This differentiation is critical because anencephaly is the predominant antenatal diagnosis to consider.^[8,15,16]^ While several craniofacial anomalies represent significant radiological differentials, researchers have observed that a comprehensive transvaginal ultrasound scan around the 12th week of gestation can effectively diagnose acalvaria in many cases.^[3,5,8,15]^

The primary treatment approach for acalvaria primarily involves conservative measures, emphasizing supportive care, and addressing any accompanying anomalies, if present.^[5]^ In some cases of scalp defects like cutis aplasia, spontaneous bone growth highlights the importance of initial conservative management for acalvaria patients. Later, skull reconstruction with bone grafting and cranioplasty is feasible, typically during school-going age.^[17]^

Generally, the prognosis for cranial vault defects is poor. Antenatal diagnosis of this fatal anomaly through careful radiological assessment is crucial. In our case, the lack of such an assessment led to a late diagnosis. Early detection can assist parents in making informed decisions regarding the continuation of the pregnancy.

## 4. Conclusion

Acalvaria is a fatal condition with a poor prognosis, often resulting in short life expectancies for affected newborns. We present a rare case of acalvaria accompanied by absent clavicles, a combination not previously documented in the literature, marking the first reported case in Palestine. Prenatal diagnosis is crucial for reducing the various burdens and impacts of this condition, especially in low-income countries. In our case, the diagnosis was delayed due to the absence of a prenatal assessment. Further research is necessary to gain a deeper understanding of the underlying causes and developmental mechanisms of acalvaria and its associated anomalies.

## Acknowledgments

We would like to thank the patient’s family for cooperating in this study.

## Author contributions

**Conceptualization:** Maaweya Jabareen.

**Data curation:** Maaweya Jabareen, Mousa Humeedat.

**Formal analysis:** Maaweya Jabareen.

**Investigation:** Asrar Alhroub.

**Resources:** Mousa Humeedat.

**Software:** Wasef Alhroub.

**Supervision:** Mosaikah Anati.

**Validation:** Bashar Qanaeer.

**Writing – original draft:** Maaweya Jabareen.

**Writing – review & editing:** Wasef Alhroub.
